# Exome genotyping, linkage disequilibrium and population structure in loblolly pine (*Pinus taeda* L.)

**DOI:** 10.1186/s12864-016-3081-8

**Published:** 2016-09-13

**Authors:** Mengmeng Lu, Konstantin V. Krutovsky, C. Dana Nelson, Tomasz E. Koralewski, Thomas D. Byram, Carol A. Loopstra

**Affiliations:** 1 Department of Ecosystem Science and Management, Texas A&M University, 2138 TAMU, College Station, TX 77843-2138 USA; 2 Molecular and Environmental Plant Sciences Program, Texas A&M University, 2474 TAMU, College Station, TX 77843-2474 USA; 3 Department of Forest Genetics and Forest Tree Breeding, Georg-August-University of Göttingen, Göttingen, 37077 Germany; 4 N. I. Vavilov Institute of General Genetics, Russian Academy of Sciences, Gubkina Str, Moscow, 119333 Russia; 5 Genome Research and Education Center, Siberian Federal University, 50a/2 Akademgorodok, Krasnoyarsk, 660036 Russia; 6 USDA Forest Service, Southern Research Station, Southern Institute of Forest Genetics, 23332 Success Road, Saucier, MS 39574 USA; 7 University of Kentucky, Forest Health Research and Education Center, 730 Rose Street, Lexington, KY 40546 USA; 8 Texas A&M Forest Service, 2585 TAMU, College Station, TX 77843-2585 USA

**Keywords:** Loblolly pine, Exome sequence capture, Target enrichment, Genotyping by sequencing, Linkage disequilibrium, Population structure, SNPs

## Abstract

**Background:**

Loblolly pine (*Pinus taeda* L.) is one of the most widely planted and commercially important forest tree species in the USA and worldwide, and is an object of intense genomic research. However, whole genome resequencing in loblolly pine is hampered by its large size and complexity and a lack of a good reference. As a valid and more feasible alternative, entire exome sequencing was hence employed to identify the gene-associated single nucleotide polymorphisms (SNPs) and to genotype the sampled trees.

**Results:**

The exons were captured in the ADEPT2 association mapping population of 375 clonally-propagated loblolly pine trees using NimbleGen oligonucleotide hybridization probes, and then exome-enriched genomic DNA fragments were sequenced using the Illumina HiSeq 2500 platform. Oligonucleotide probes were designed based on 199,723 exons (≈49 Mbp) partitioned from the loblolly pine reference genome (PineRefSeq v. 1.01). The probes covered 90.2 % of the target regions. Capture efficiency was high; on average, 67 % of the sequence reads generated for each tree could be mapped to the capture target regions, and more than 70 % of the captured target bases had at least 10X sequencing depth per tree. A total of 972,720 high quality SNPs were identified after filtering. Among them, 53 % were located in coding regions (CDS), 5 % in 5’ or 3’ untranslated regions (UTRs) and 42 % in non-target and non-coding regions, such as introns and adjacent intergenic regions collaterally captured. We found that linkage disequilibrium (LD) decayed very rapidly, with the correlation coefficient (*r*^2^) between pairs of SNPs linked within single scaffolds decaying to half maximum (*r*^2^ = 0.22) within 55 bp, to *r*^2^ = 0.1 within 192 bp, and to *r*^2^ = 0.05 within 451 bp. Population structure analysis using unlinked SNPs demonstrated the presence of two main distinct clusters representing western and eastern parts of the loblolly pine range included in our sample of trees.

**Conclusions:**

The obtained results demonstrated the efficiency of exome capture for genotyping species such as loblolly pine with a large and complex genome. The highly diverse genetic variation reported in this study will be a valuable resource for future genetic and genomic research in loblolly pine.

**Electronic supplementary material:**

The online version of this article (doi:10.1186/s12864-016-3081-8) contains supplementary material, which is available to authorized users.

## Background

Southern forests dominated by pines contain one third of the entire forest carbon in the contiguous U.S. [1]. Among the southern pines, loblolly pine is the most common, productive and valuable commercial timber species due to its rapid growth and vast territory, comprising 80 % of the planted forestland and over one half of the standing volume in the southern U.S. The native range of loblolly pine extends south from New Jersey to central Florida, and west to central Texas, occupying 55 million acres of forest land [2, 3]. Since forests capture and store carbon dioxide through photosynthesis, the widely planted loblolly pine in the southern U.S. provides great value in offsetting atmospheric carbon dioxide and mitigating climate changes caused by greenhouse gas emissions [4, 5].

Genomic tools and resources that focus on the dissection of complex traits are revolutionizing traditional loblolly pine breeding and assist with the breeding and deployment of genotypes better adapted to climate change and able to sequester greater amount of carbon. Two key prerequisites for development and application of genomics-assisted breeding are the characterization of the genetic variation and the collection of genome-wide molecular markers. A high level of genetic polymorphism is expected in loblolly pine due to its life traits, typical for conifer species, such as longevity, wide geographic distribution, large effective population size and high outcrossing rate. This was confirmed in early studies with isozymes [6, 7], DNA-based markers [8–10], and especially more recently with SNP [11–13] markers. About 4000 SNP markers have been genotyped in the previous association genetics studies [11, 13, 14], but many more markers are needed for genomic selection [15–18].

In the previous loblolly pine association mapping studies, an Illumina Infinium high-throughput SNP genotyping array developed for multiplex genotyping of 7216 SNP markers was used to dissect genetic control of diverse phenotypic traits [11, 13, 14, 19–21]. These SNPs were derived originally from amplicon sequencing data based on a relatively small, but range-wide sample of 18 loblolly pine megagametophytes and using PCR primers that were designed using unigene contig sequences assembled from expressed sequence tag (EST) sequences. Finally, about 4000 SNPs from this 7 K SNP array were polymorphic or could be genotyped in follow-up studies [11, 13, 14, 19–21].

Given adequate geographic distribution sampling, the genetic structure underlying loblolly pine populations could also be elucidated using SNPs. For instance, Eckert et al. [19] analyzed SNP and simple sequence repeat (SSR) markers among 907 rangewide loblolly pine trees and found that the population structure reflected mainly the Mississippi River discontinuity.

Efficiency of marker-assisted breeding and genomic selection depends largely on genome wide linkage disequilibrium (LD). Brown et al. [12] found substantial historic recombination between SNPs in the sampled alleles sequenced in 19 genes and demonstrated that LD significantly declined within 2 Kb in loblolly pine. A genome wide study by Chhatre et al. [11] confirmed rapid LD decay in loblolly pine. These studies suggested that a very large number of markers would be required to link phenotypes to genotypes in association mapping studies and in genomic selection of this species. Therefore, for a species such as loblolly pine with a large genome and rapid LD decay, even thousands of markers cannot meet the requirement of identifying all important functional genomic regions. Fortunately, genotyping by sequencing (GBS), which enables simultaneous marker discovery and genotyping, has facilitated the generation of large numbers of molecular markers [22]. Nevertheless, the large size and complex structure of the loblolly pine genome pose challenges for the whole genome resequencing. The loblolly pine genome assembly v. 1.01 spans 23.2 Gbp and contains 14.4 million scaffolds [23]. Tentatively, 50,172 putative genes with an average length of 2.7 Kbp have been annotated in the current loblolly pine genome assembly [24]. Moreover, various highly repetitive DNA elements compose up to 82 % of the loblolly pine genome, among which retrotransposons dominate and comprise 62 % of the genome [23, 24]. Therefore, reduction of genome complexity is highly desired for application of GBS to loblolly pine.

In our study, we used the entire exome region for target enrichment to limit GBS to mostly coding regions, which represent only ~40–60 Mbp of sequence space or less than 0.2 % of the entire loblolly pine genome. In the previous studies, technologies for solution-based enrichment of target regions of interest have been developed for loblolly pine [25–27]. Capture size has been significantly expanded due to the improvement in probe design and capture efficiency, making it possible to capture up to 200 Mbp of target sequence with a single design (NimbleGen SeqCap EZ Developer Enrichment Kit). These developments made it possible for us to target and enrich the entire loblolly pine exome, thus greatly enlarging the available number of molecular polymorphisms in loblolly pine.

**Fig. 1:**
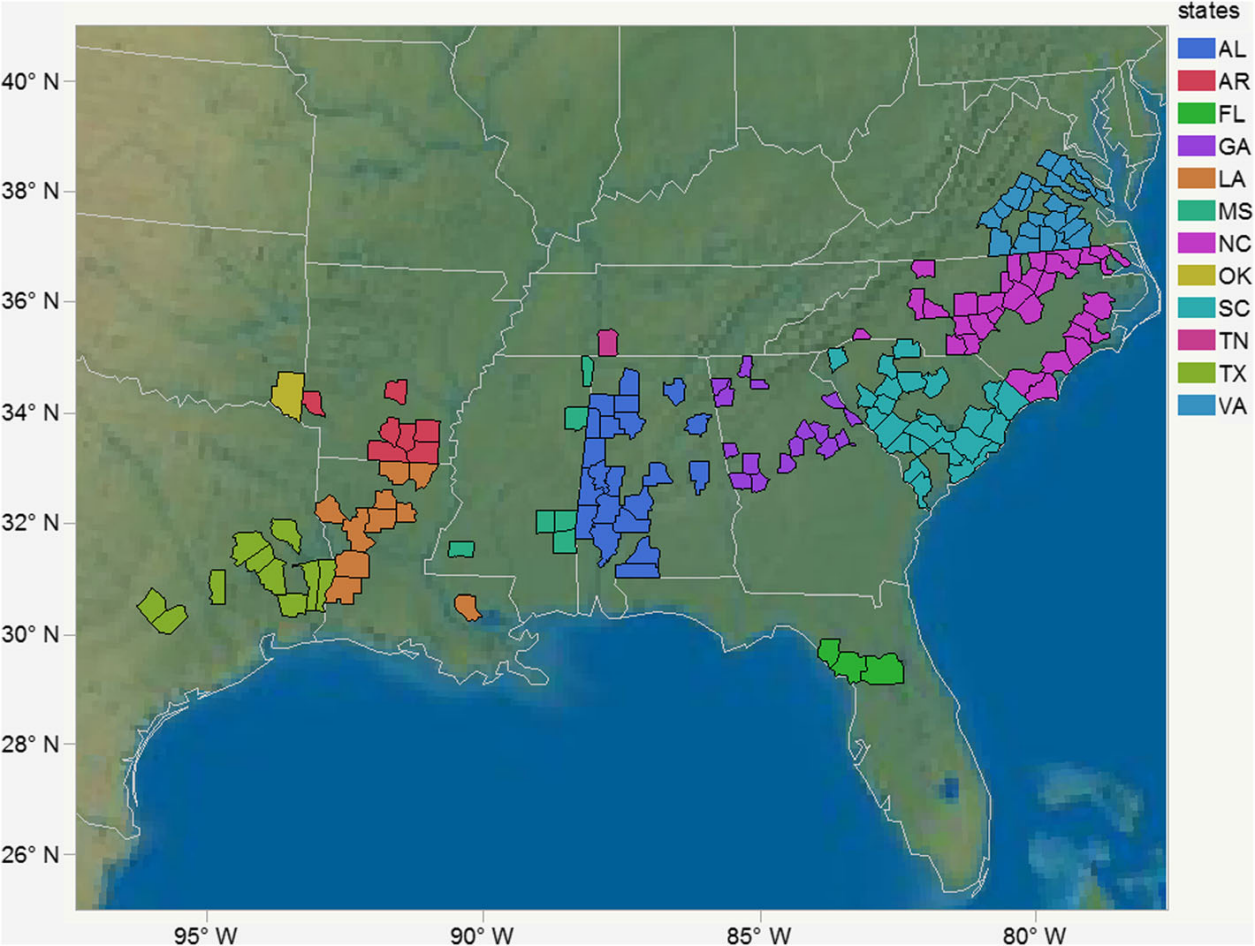
The counties of origin of the maternal trees colored by states. This map shows the sampling sites of the 362 out of 375 maternal parents of the ADEPT2 population used in this study

In this study, we describe the probe design and efficiency of the loblolly pine exome capture using the NimbleGen SeqCap EZ method in a population sample containing 375 clonally-propagated trees from an association mapping population generated for the Allele Discovery of Economic Pine Traits II (ADEPT 2) project [14]. Counties of origin are known for 362 out of 375 maternal trees (Fig. 1). SNPs were identified by aligning the exome capture sequences to loblolly pine genome assembly v. 1.01 [28]. The inferred SNP genotypes were then applied to study LD decay and population structure.

## Results and discussion

### Exome target enrichment hybridization probe design and assessment

Sequence capture oligonucleotide probes were designed using 199,723 exons in 48,391 (34,059 full-length and 14,332 partial-length) high quality tentative genes listed in gene annotation v. 2.0 for loblolly pine genome assembly v. 1.01 [24]. The final probe set used in this study is available from Roche NimbleGen as custom SeqCap EZ design “140422_Ptaeda_Exome_ML_EZ_HX3”. Approximately 2.1 million single strand oligonucleotide probes were designed and produced in total that covered 90.2 % (46,206,684 bp) of the target regions. The regions not covered (gaps) were areas where the probe selection algorithm could not find a valid probe. These gaps usually represented repetitive DNA regions that, if included, could be expected to cause problems by capturing other homologous regions in the genome and, therefore, decrease capture and mapping efficiency.

In the first published study of exome capture in loblolly pine, 54,773 probes representing 6.57 Mbp of target exome were designed using 14,729 unique transcripts derived from the assembly of ESTs [26]. However, the unavailability of a reference genome and, therefore, lack of information on the exon-intron boundaries, negatively affected the probe design. This caused insufficient capture and cross-hybridization and decreased the capture efficiency. This problem was mitigated in our exome capture study, because the probe set covered almost the entire exome and its design took into account the exon-intron structure. The designed probe set covered ~46 Mbp of target exome and included previously uninvestigated genomic regions. The risk of capturing pseudogenes was decreased by using only genes classified as “high quality” to design the probes. A key concern during the probe design was the exclusion of those probes that might cross-hybridize with non-target regions and repetitive elements, especially considering that 82 % of the loblolly pine genome consists of the highly repetitive sequences [23]. In this study, the preliminary probes were stringently filtered to exclude possible cross-hybridization with non-target regions and repetitive elements. Although the capture size could be potentially expanded, if the filtering criteria had been relaxed, the stringent filter guaranteed the hybridization specificity and prevented cross-hybridization.

### Exome capture sequence alignment and efficiency

We multiplexed ten individually amplified and uniquely barcoded trees per library for capture hybridization, enrichment, and sequencing. After demultiplexing and filtering, we obtained between 25.25 and 60.55 million sequence reads per tree. The reads of each tree were mapped to loblolly pine genome assembly v. 1.01 [23, 24, 28]. Nearly 99 % of the sequence reads were mapped to the reference genome assembly. In order to improve the SNP discovery accuracy, the mapped reads were further filtered and only the uniquely mapped, properly paired (correctly oriented with respect to one another) and non-redundant reads were used for downstream analyses. After filtering, 62–75 % of the total reads (71 % per tree on average) were used for SNP calling (Additional file 1: Table S1).

**Fig. 2:**
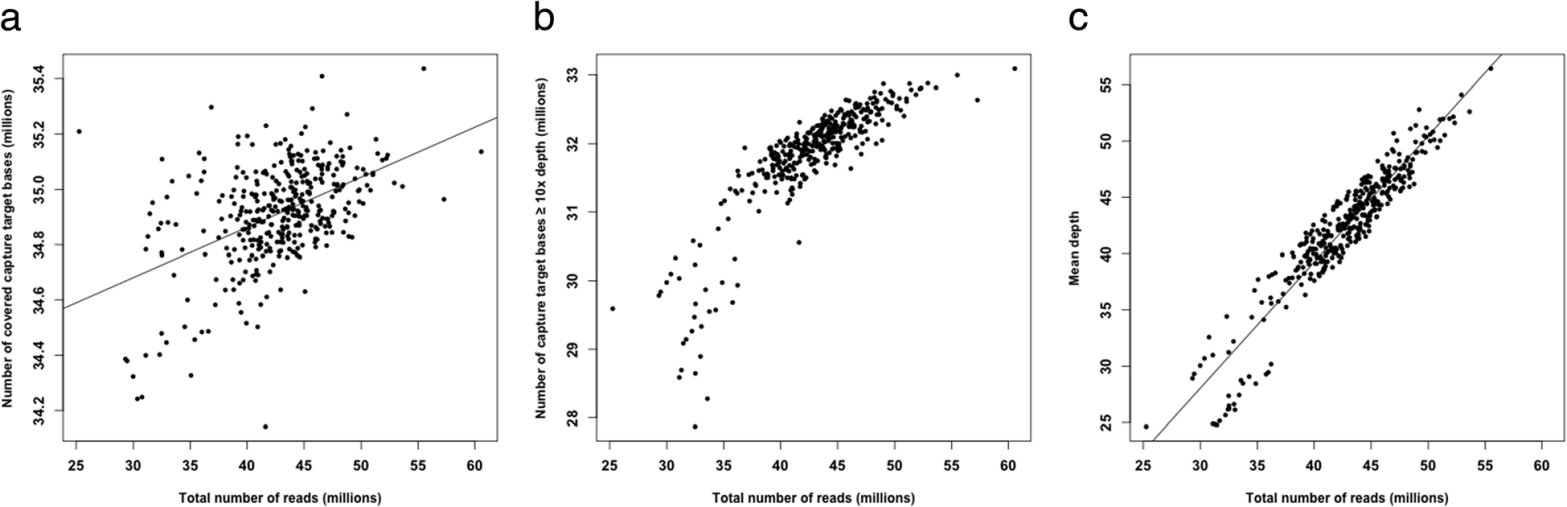
Relationship between reads and capture target bases. **a** Relationship between reads and numbers of covered capture target bases. The numbers of captured target nucleotide bases are plotted against total number of sequence reads obtained in 375 trees from exome capture sequencing. The linear regression coefficient (*r* ^*2*^) is 0.23 (*P* < 0.001). **b** Distribution of on-target coverage ≥ 10x depth across the 375 trees. The numbers of capture target bases with a coverage depth of ten or greater sequence reads per target are plotted against the total number of sequence reads. The relationship seemed approximately linear within a limited range of the total number of reads at 37–55 million. **c** Distribution of mean coverage depth across the 375 trees. The mean coverage depth is plotted against the total number of sequence reads. The linear regression coefficients (*r* ^*2*^) was significant (*P* < 0.001) and equalled 0.72

Capture breadth and depth were investigated to examine capture efficiency and target specificity. For the uniquely mapped, properly paired, and non-redundant reads for each tree, we calculated the number of reads that mapped to the capture target regions using the BEDtools software v. 2.23.0-20-gada04b6 [29]. On average, 67 % of the reads per tree (59–74 %) mapped to the capture target regions. Additional non-target captured sequences included those adjacent to target or homologous regions. Between 91 and 95 % of the capture target regions were covered by at least one read. The number of covered capture target bases was weakly and positively correlated with an increase in sequencing output (Fig. 2a; *r*^*2*^ = 0.23, *P* < 0.001).

**Fig. 3:**
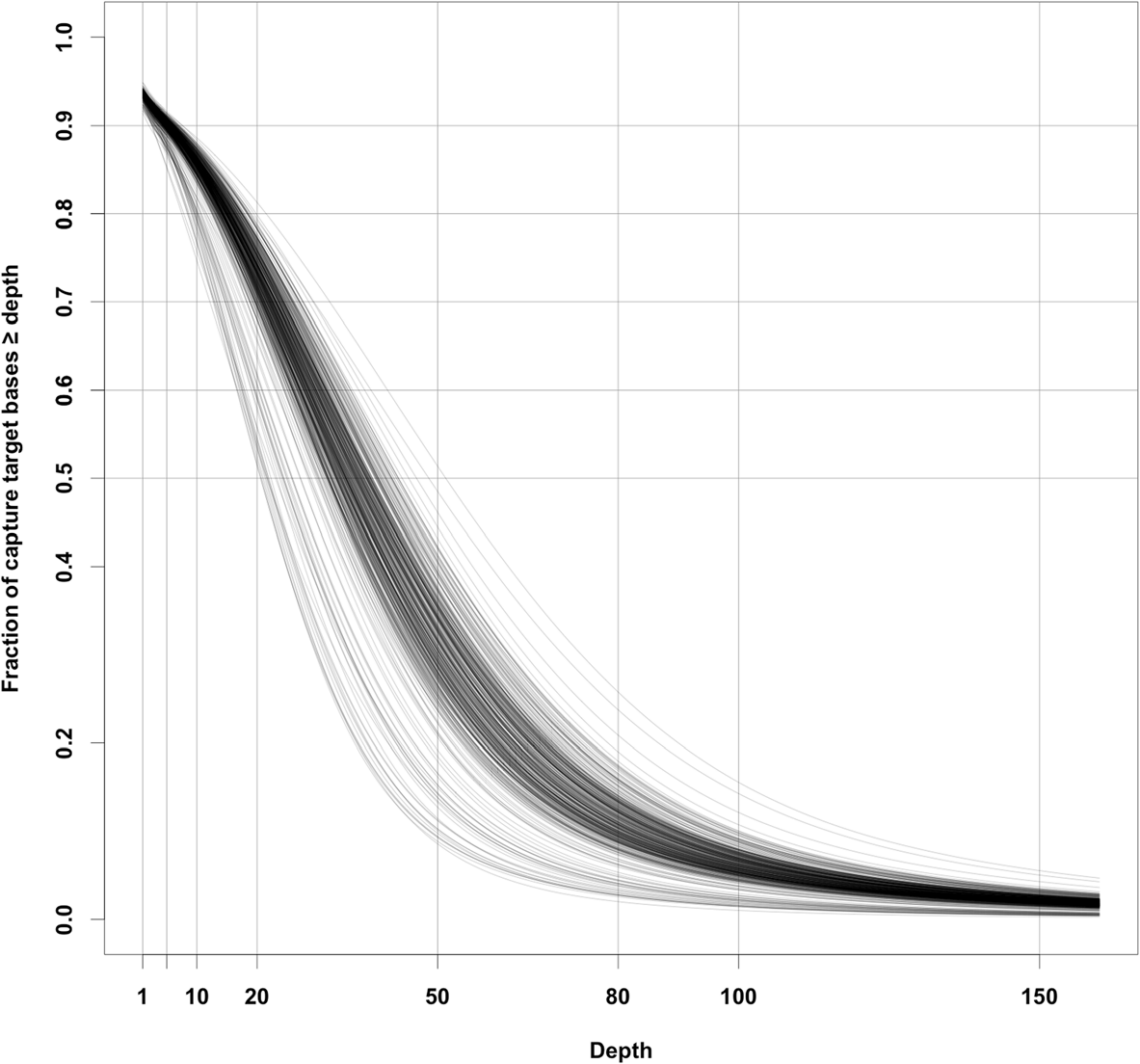
Cumulative distribution of coverage depth of captured target bases in 375 trees. Each line represents a single tree

Coverage depth among the 375 trees was generally uniform and it was consistent across target regions. Among all the trees, at least 83 % of the capture target bases had coverage of 5X, 72 % - 10X, and 49 % - 20X (Fig. 3). The number of target bases with coverage depth of 10X or greater (Fig. 2b) seemed to change approximately linearly within a limited range of the total number of reads at about 37–55 million. Below this range, the number of captured bases increased faster than within the range. But the effect of increasing became weaker above 55 million. The mean coverage depth (Fig. 2c) increased linearly as the total sequencing output increased (*r*^*2*^ = 0.72, *P* < 0.001), although the variance seemed slightly increased for the lower numbers of the total number of reads.

Multiplexing individually and uniquely indexed samples before capturing and sequencing greatly saves time and money and has become a standard procedure in sequence capture experiments. However, sufficient sequencing depth (output) is still needed to guarantee a higher coverage depth on the target regions. Fig. 2b and c demonstrate that the coverage depth is positively correlated with the sequencing output. Therefore, multiplexing should be reasonable and should ensure sufficient on-target coverage depth to avoid problems associated with low SNP detection power. In our study, an uneven number of sequencing reads across different individual tree samples could be mainly due to multiplexing of unequal amounts of the sample libraries.

Some of the reads could not be mapped to the reference genome, likely due to either incomplete assembly of the reference genome or multiple sequencing errors in the reads that exceeded the mismatch tolerance threshold of the mapping parameters. Although the probes were filtered for cross-hybridization prior to the actual hybridization step, further filtering of the multi- and improperly mapped reads was important in order to retain only the high quality mapped reads for downstream analyses. Similarly, the redundant reads were also filtered to remove the potential PCR duplicates and to correct the coverage depth.

The read mapping results demonstrated a high level of on-target efficiency in this research. This guarantees the target regions have enough coverage depth. Less than 9 % of the target regions had no matching reads. The main reason for this was that the probes covering these regions were filtered out to avoid cross-hybridization. It should also be noted that the current reference genome assembly is still under development and the target regions with no matching reads could potentially be artifacts or mis-assembled parts of the reference genome.

### Single nucleotide polymorphism (SNP) discovery

SNPs were detected in 375 individual trees using the SAMtools software v. 1.1 [30]. The raw SNPs were filtered using the selection criteria of being bi-allelic sites with at least 10X sequencing depth in at least 90 % of the individuals, and with the minor allele frequency (MAF) ≥0.05. A total of 972,720 SNPs were acquired for downstream analyses. These SNPs were located in 38,702 scaffolds of the loblolly pine reference genome assembly v. 1.01. A maximum of 854 SNPs were detected in one scaffold. Based on annotation of genomic regions, most of the identified SNPs resided in exons, but some resided in introns or unclassified regions. Among all the SNPs, 58 % were located in exons with an average SNP density of 11.5 SNPs/Kbp (one SNP per 87 bp); 53 % were located in coding regions (CDS); 2 % in five prime untranslated regions (5’ UTR); 3 % in three prime untranslated regions (3’ UTR) and 13 % in introns. By position relative to capture target region, 51 % of all SNPs were located in capture target regions with an average SNP density of 13.2 SNPs/Kbp (one SNP per 76 bp), and 49 % were located in off-target regions (Table 1). The number of SNPs detected in exons was more than in on-target regions because the capture extended to the adjacent area of each target.

**Table 1:** Number and percent of 972,720 SNPs located in different genomic regions

Category	SNPs	Percent
Exon	564932	58.08
CDS	513652	52.81
5’ UTR	17693	1.81
3’ UTR	33587	3.45
Intron	127863	13.14
Unclassified	279925	28.78
On-target	498451	51.24
Off-target	474269	48.76

One of the most important goals of exome sequencing is to identify the genetic variants that can be used in the association mapping analysis to dissect the phenotypes of interest. Such analyses require high quality SNPs, and therefore we focused only on those SNPs, both within and outside of exons, that passed the strict filtering criteria described above.

### Population genetics metrics

SNPs with a MAF less than 0.05 were excluded, therefore SNP allele frequencies ranged between 0.05 and 0.5 with a median of 0.14 (Additional file 2: Figure S1). The average transition to transversion ratio (T_S_/T_V_) was 1.96 over all regions (Table 2, Additional file 3: Table S2). This value was higher in CDS than in UTRs. The transition bias could be attributed to natural selection on the nonsynonymous transversion, and the even higher ratio for CDS could be caused by the increased presence of methylated cytosine in CpG dinucleotides where the methylated cytosine can easily undergo deamination and transition to a thymine [31].

**Table 2:** Transition to transversion ratios (T_S_/T_V_) for 972,720 SNPs categorized in different genomic regions

Total	CDS	Exon	5’ UTR	3’ UTR
1.96	1.98	1.93	1.58	1.45

**Fig. 4:**
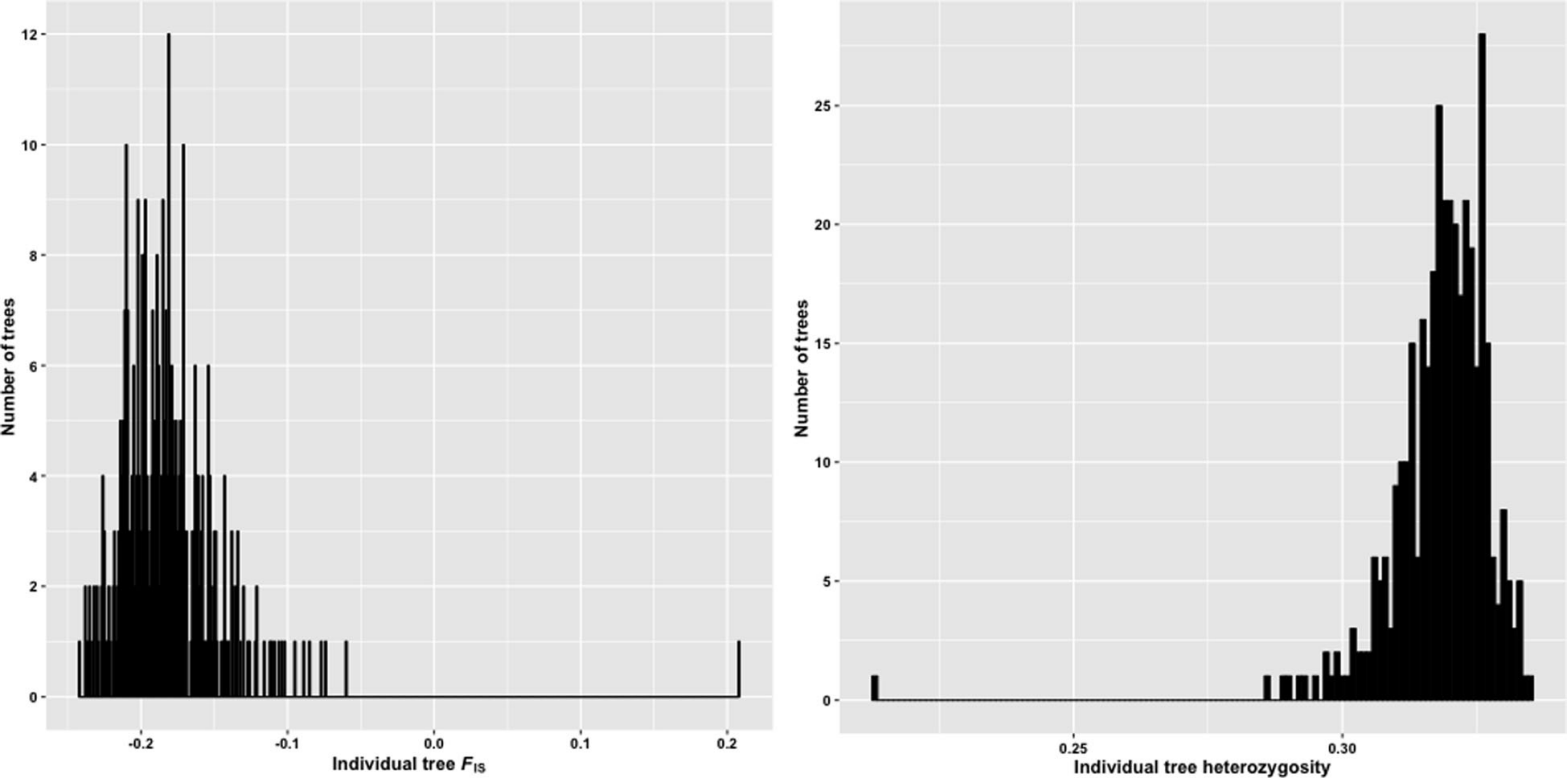
*F*_IS_ (*left*) and heterozygosity (*right*) distributions among 375 trees

Heterozygosity and *F*_IS_ were estimated on an individual basis (Fig. 4). The results indicated a low inbreeding rate and a high level of genetic diversity. Among all individuals, the *F*_IS_ values were generally below zero, ranging between −0.24 and −0.06, except in tree 634A, where it was 0.21. Heterozygosity was between 0.29 and 0.33 except in 634A, where it was 0.21. These values were expected because loblolly pine is a highly outcrossing and polymorphic species. In addition, the ADEPT2 population was established for association mapping with presumably unrelated trees originally sampled from across a wide part of the natural range. Tree 634A may be a progeny from selfing or a mating between closely related trees.

After Bonferroni correction (adjusted *P*-value < 5e-8), 188,072 (19 %) out of 972,720 SNPs significantly departed from Hardy-Weinberg equilibrium (HWE). Nucleotide diversity (π) in different genomic regions was estimated in a sliding window of 50 bp with a step of 25 bp (Additional file 4: Table S3). Regions out of annotated genes had higher average nucleotide diversity than in annotated genes. This could be due to selection constraints. However, it should be noted that the highly diverged sequences could not map to the reference genome, hence biasing the diversity estimates.

### Genome-wide linkage disequilibrium (LD)

**Fig. 5:**
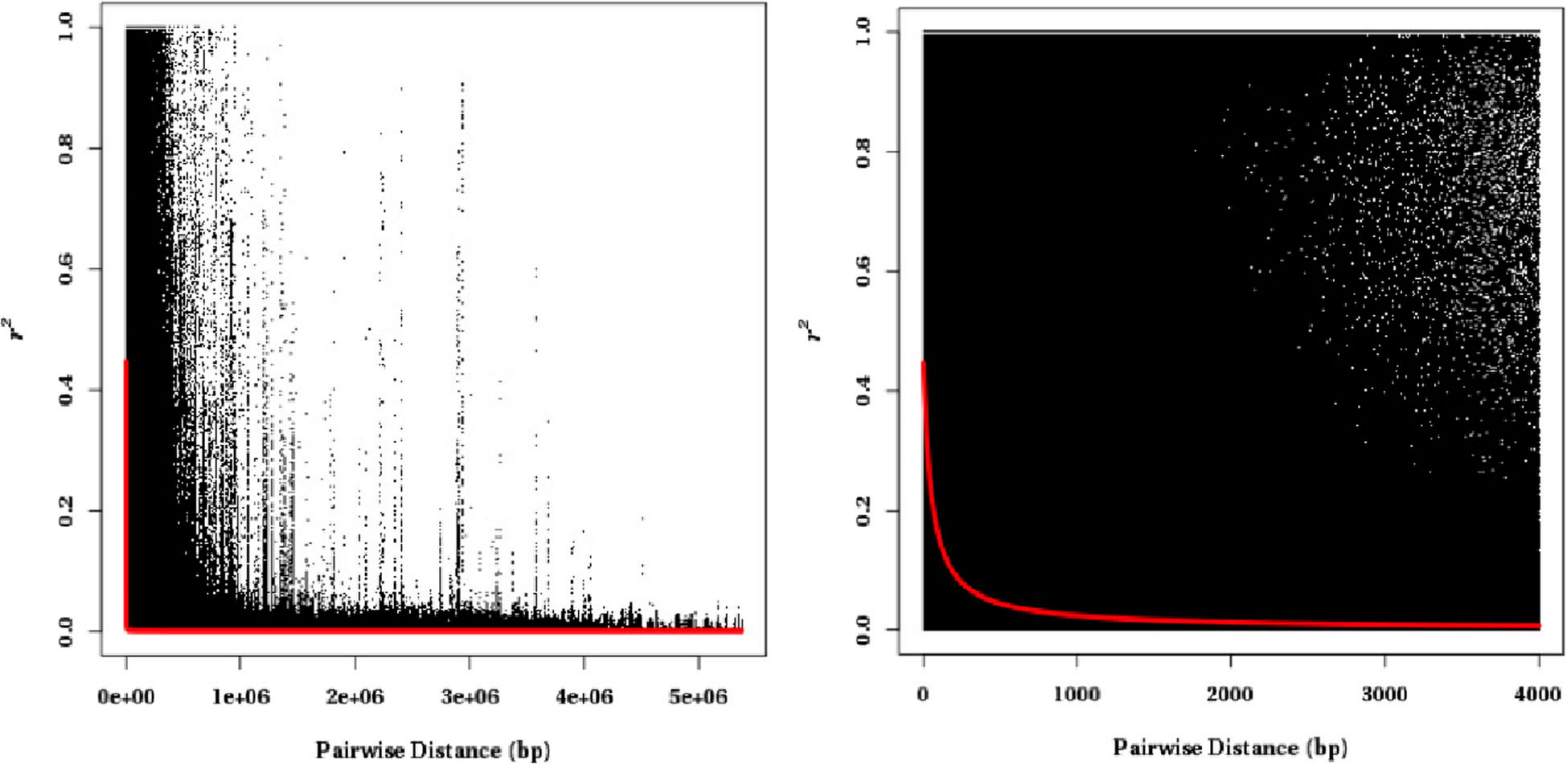
Linkage disequilibrium decay plot for 375 trees based on 972,720 SNP markers. Pairwise linkage disequilibrium coefficients (*r* ^*2*^) calculated for all 375 trees were plotted against the physical distances (bp) between all pairs of SNPs within the same scaffolds (*left*) and between pairs of SNPs within the same scaffolds located within 4000 bp (*right*). The trendlines of the nonlinear regressions (*r* ^*2*^) against physical distance between the SNPs are indicated in *red*

LD is a non-random association of alleles at different loci and may indicate the genetic forces that structure the genome [32]. Investigations of genetic diversity and LD are prerequisites for association mapping and help in interpretation of results. We calculated the zygotic LD (squared correlation coefficient *r*^*2*^) values for all SNP pairs within each scaffold in the genome assembly and plotted them against the physical distances between the same SNP pairs in the scaffold (Fig. 5). The average LD for linked SNPs was inferred from the trendlines of the nonlinear regressions and started from 0.44, then decayed by half (0.22) at 55 bp, to 0.10 at 192 bp, and to 0.05 at 451 bp. The proportion of SNP pairs located within the same scaffold with *r*^*2*^ > 0.1 was 18 % in this population, and with *r*^*2*^ > 0.8 it was 3 %.

Highly outcrossing conifers are expected to have a rapid LD decay. Neale and Savolainen [33] reported that the *r*^*2*^ decayed to less than 0.20 within ~1500 bp based on 19 candidate genes in loblolly pine. In spruces, LD displayed diverse patterns among different genes or the same genes in different species, declining rapidly to half between a few base pairs and 2000 bp [34]. In Douglas-fir (*Pseudotsuga menziesii*), LD decayed > 50 % over relatively short segments from *r*^*2*^ = 0.25 to 0.10 within 2000 bp based on sequencing 18 genes [35]. LD estimates in this study based on the exome-derived sequences indicated an even faster decay than previously reported. This could be due to the much larger number of gene regions analyzed in this study. The discrepancies can be partly explained also by different methods used for estimating LD. The abovementioned studies calculated gametic LD statistics *r*^*2*^ using megagametophyte haplotypes, while in this study, zygotic LD between genotypes was calculated. However, gametic LD can also be calculated in our study based on the inferred (phased) haplotypes. When we used the phased haplotypes inferred by the software Beagle v. 4.1 [36] for the 972,720 SNPs to calculate gametic LD, a slower decay was observed, with LD decaying by half (*r*^*2*^ = 0.22) at 79 bp and to *r*^*2*^ = 0.10 at 280 bp. The rate of LD decay can vary between genes and across different genome regions [34]. Therefore the generality of LD distribution across the entire loblolly pine genome remains to be further analyzed because only a relatively small and highly specific part of the entire genome was studied here. Our study relied also on the accuracy of contig and scaffold assembly in the draft reference genome that should be verified and ordered in the future studies.

### Population structure

Evaluation of population structure is crucial for association mapping. If not accounted for, population structure may cause spurious associations between markers and phenotypes [37]. The ADEPT2 population trees included in this study were the clonally-propagated, open-pollinated progeny of the originally sampled trees. The maternal origins were known for 362 out of 375 trees. The 362 trees can be divided into two sub-samples based on the geographic location of their maternal parents: 1) the sub-sample west of the Mississippi River represented by 55 trees from four states, and 2) the sub-sample east of the Mississippi River represented by 307 trees from eight states. *F*_ST_ was estimated on a per-site basis following Weir and Cockerham [38]. The *F*_ST_ range was between -0.01 and 0.72, with a median of 0.0087 (Distribution of *F*_ST_ values across all loci is presented in Additional file 5: Figure S2). The mean *F*_ST_ was 0.026, and the weighted *F*_ST_ was 0.028. Generally, the genetic differentiation between these two sub-samples was relatively low, but statistically significant.

We then applied the software fastStructure [39] to infer the admixture proportion using our genotyping data. We thinned the marker set to no more than a single marker within 1 Mbp on each scaffold, which resulted in a presumably unlinked set of 30,146 SNPs. After testing a number of potential subpopulations (clusters) with fastStructure, ranging from *K* = 1 to *K* = 12 (where *K* is the number of subpopulations or clusters), we ran the recommended fastStructure algorithm for multiple *K* to choose the appropriate number of model components that explained structure in the dataset. The output showed model complexity that maximized marginal likelihood when *K* = 2, and the model components used to explain structure in data when *K* = 7. Therefore, we considered two and seven clusters as the most likely subpopulation clustering explaining the relationship between admixture proportion and geographical sites.

**Fig. 6:**
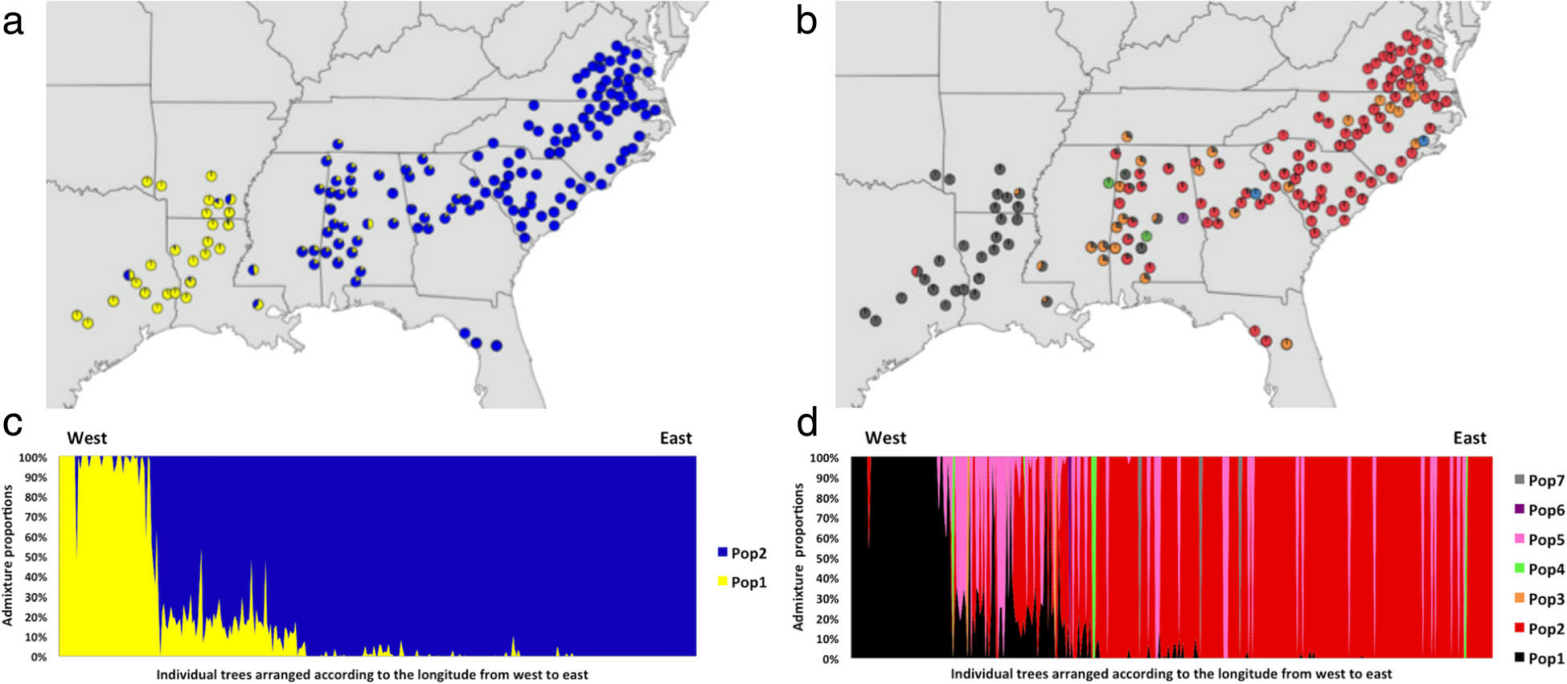
Summarized admixture proportion distributions for *K* = 2 and *K* = 7. **a** & **b** Summarized admixture proportions plotted on the map. Each pie chart is partitioned via summarized population assignments inferred by fastStructure. **c** & **d** Individual tree admixture proportion distributions. The trees are aligned on the x-axis according to the longitude from west to east

A clear geographical trend could be observed when the admixture proportions of each tree across clusters were plotted on a map (Fig. 6a and b). The segment in each pie chart corresponds to the summarized population assignment inferred by the software. We further aligned the admixture proportion of each tree with the longitude from west to east (Fig. 6c and d). Strong statistical correlations were observed between longitude and admixture proportion (*r*^*2*^ = 0.75 when *K* = 2 and *r*^*2*^ = 0.74 when *K* = 7). In Fig. 6c and d, vertical lines arranged from left to right correspond to the individual trees according to their original maternal parents’ geographic locations from west (Texas) to east (North Carolina) in the southeastern U.S. Each vertical line represents admixture proportions for an individual tree partitioned when *K* = 2 (Fig. 6c) or *K* = 7 (Fig. 6d). The left 55 trees on the X-axis represent the trees west of the Mississippi River, while the other trees are from east of the Mississippi River.

It has been widely recognized that the glacial advance and retreat have altered the landscape of the Mississippi Valley and the species became restricted into glacial refugia, thus high dissimilarity was formed between refugia populations [40]. A postglacial barrier to dispersal was created between populations located west and east of the Mississippi River and thus decreased the gene exchange and increased the overall genetic variance in some species [40, 41]. The discontinuity is also evident in loblolly pine, as can be concluded from genetic differentiation estimated in our study based on ADEPT2 population, and in the earlier studies that were based on limited numbers of SNP and SSR markers [19, 42].

## Conclusions

Our results demonstrated the efficiency of exome capture for genome-wide genotyping of a species with a large, complex genome. We took advantage of target sequence capture technology as well as the recently released draft loblolly pine reference genome assembly and annotation to design the exon specific probes across a 49 Mbp target region. The capture efficiency and specificity were high, paving the way for reliable SNP calling. In total, 972,720 SNPs were detected from exon associated sequences in an association mapping population ADEPT2 that included clones of 375 loblolly pine trees originally sampled across a wide range. This population is highly heterozygous and consists of two distinct subpopulations (genetic clusters), west and east of Mississippi River, respectively. LD decayed faster than previously reported suggesting that a great amount of SNPs will be required for association mapping. The highly diverse genetic variation reported in this study provides a valuable resource for loblolly pine breeding through marker-assisted selection and genomic selection. Further research, including genome wide association studies and functional analyses of candidate genes, is now possible and will contribute molecular tools for selection of loblolly pine genotypes adapted to changing climate scenarios.

## Methods

### Plant material and genomic DNA extraction

The population studied here was from the ADEPT2 project [14]. Maternal parents of the ADEPT2 population were originally sampled across 12 states in the southeastern U.S., extending from Virginia to Florida, and west to central Texas (Fig. 1). Seeds were collected from the maternal trees after open pollination. Trees were grown from open-pollinated seeds for 1 year and then were hedged and established for use in the ADEPT2 project. In the spring of 2010, rooted cuttings from 384 trees (i.e., clones) of the ADEPT2 population were established at the Harrison Experimental Forest at the Southern Institute of Forest Genetics, near Saucier, Mississippi. Needle samples were collected from 375 surviving clones for extraction of genomic DNA in June 2012 and stored at -20 °C. Four needles from each sample were ground in liquid nitrogen to a fine powder. DNA was extracted using QIAGEN DNeasy Plant Mini Kits following the standard protocol except in the last step, where 1 × TE buffer with low EDTA was used for elution. Genomic DNA samples with OD260/OD280 ratios between 1.7 and 2.0 without signs of degradation were used for downstream applications.

### Probe design

Probes were designed using Gene Annotation v. 2.0 for loblolly pine genome assembly v. 1.01 [23, 24]. We submitted the 49,216,700 bp of sequence that represented 199,723 exons to Roche NimbleGen Inc. for sequence capture probe selection. The target regions were inferred using the exon coordinates available in the files “ptaeda.v1.01 scaffolds.trimmed.all.genes.highq_whole.gff3”, which included annotation for 34,059 full length, high quality genes, and “ptaeda.v1.01 scaffolds.trimmed.all.genes.highq_partial.gff3”, which included 14,332 partial length, high quality genes. Exons shorter than 100 bp in length were extended (padded) to 100 bp. After screening, a total of 196,068 exons (51,239,342 bp) were selected for probe design. A relatively conservative threshold was used to design unique probes that could tolerate no more than five single-base indel or single nucleotide substitution mismatches with the genome. The length of the probes varied between 50 and 100 bp. The average length was 76.5 ± 4.2 bp, with a median of 76 bp.

### Sequencing library preparation and target enrichment

Each genomic DNA was diluted to 25 ng/μl in 1 × TE buffer with low EDTA and 50 μl of each DNA solution was fragmented to have an average size distribution of ~180–220 bp using a Covaris sonicator. KAPA Library Preparation Kits (Illumina® Platforms) were used to construct a library for each DNA sample. After post-ligation cleanup and dual-SPRI size selection, the sample libraries were amplified and checked for quality and quantity using the Agilent 2100 Bioanalyzer and PicoGreen dsDNA quantitation assays. The amplified sample library was acceptable if the OD260/OD280 ratios were between 1.7 and 2.0, respectively, the yield was more than 1.0 μg, and the average fragment size was between 150 and 500 bp.

The Roche NimbleGen SeqCap EZ system was used for hybridization and target enrichment. Briefly, equal amounts of each of ten libraries representing uniquely individually indexed and amplified trees were mixed in a single exome enrichment and sequencing pool with a combined mass of at least 1.25 μg. The multiplexed paired-end sequencing libraries were hybridized with the target sequence capture probes and the mixture was incubated at 47 °C for 72 h. After wash and recovery steps, the captured multiplex DNA samples were amplified and purified. Following quality check, the captured multiplex DNA samples were loaded into Illumina HiSeq 2500 flowcells (one exome enriched pool of 10 original sample libraries per a single flowcell lane) and sequenced using 2 × 125 cycles at the Texas A&M University Genomics and Bioinformatics Service (College Station, Texas, USA).

### Sequence read alignment and analysis

Sequence reads for each of the 375 trees were filtered and demultiplexed. Then, the reads were mapped to loblolly pine genome assembly v. 1.01 [23, 24, 28] using the “mem” routine in the BWA software v. 0.7.12 [43] with the default parameters. The SAM files were converted to BAM files using the “view” routine in the SAMtools software v. 1.1 [30]. The “flagstat” routine in the SAMtools software was applied to calculate the mapping percentage of reads. The reads were filtered by the “view” and “sort” routines in the SAMtools software to acquire only the uniquely mapped and properly paired sorted reads. The “rmdup” routine in the SAMtools software was used to remove potential PCR duplicates from the filtered reads. The “intersect” routine in the BEDtools software v. 2.23.0-20-gada04b62.18 was applied to estimate the percentage of reads on target regions and the “coverage” routine was applied to visualize coverage of targeted DNA [29].

Raw SNPs were called using the “mpileup” routine in the SAMtools software with 20 as the minimum mapping quality threshold for an alignment. The raw SNPs were filtered for downstream analyses, and only those that met the following criteria were kept: 1) 10× sequencing coverage in no less than 90 % of all individuals. 2) bi-allelic; 3) minor allele frequency greater than 0.05. The VCFtools v. 0.1.12b software [44] was applied to classify the SNPs according to their genomic regions and their positions relative to capture target regions. The SNP density was determined as the number of SNPs in a given region divided by the length of that regions.

### Population genetics metrics

The VCFtools software was applied to calculate the minor allele frequency (MAF), the ratio of transition to transversion (Ts/Tv), individual heterozygosity and *F*_IS_, and nucleotide diversity. The histogram graphs were plotted using the ggplot2 v. 2.1.0 package in R v. 3.2.3 [45, 46]. The squared correlation coefficient between genotypes (*r*^*2*^) on the same scaffold was used as an LD measure and calculated using the “geno-r2” routine in the VCFtools software. The trendline of LD decay along physical distance were fitted by nonlinear regression following Hill and Weir [47]. R software was applied to display the results [46]. The *F*_ST_ was estimated using the “weir-fst-pop” routine in the VCFtools software.

The SNP set was thinned to a single marker within every 1 Mbp distance in each scaffold” and converted to the PLINK software format using the “thin” and “plink” routines in the VCFtools software. The PLINK format was further converted to the PLINK BED format using the “make-bed” routine in the PLINK software v. 1.9 [48]. The fastStructure software with the simple prior was applied to infer the most likely population structure by testing different number of potential subpopulations or clusters (*K*) from 2 to 12 [39]. The recommended algorithm incorporated in fastStructure was applied to determine the reasonable choice of K. The admixture proportions of each individual were plotted using the Excel and R v. 3.2.3 [46].

## Additional files

Additional file 1: Table S1. Mapping statistics of exome capture derived sequence reads for 375 loblolly pine trees. (XLSX 44 kb) Additional file 2: Figure S1. Minor allele frequency (MAF) distribution among 972,720 SNPs. (PDF 53 kb) Additional file 3: Table S2. Transition (T_S_) and transversion (T_V_) nucleotide substitutions summary. Numbers of T_S_ and T_V_ for 972,720 SNPs in different genomic regions. (PDF 51 kb) Additional file 4: Table S3. Nucleotide diversity (*π*) estimated in a sliding window of 50 bp with a step of 25 bp in different genomic regions. (PDF 59 kb) Additional file 5: Figure S2.
*F*_ST_ distribution across all loci. The range is between -0.01 and 0.72, with a median of 0.0087. The mean *F*_ST_ is 0.026, and the weighted *F*_ST_ is 0.028. (PDF 59 kb)

## Abbreviations

CDS: Coding sequence
EST: Expressed sequence tag
GBS: Genotyping by sequencing
LD: Linkage disequilibrium
SNP: Single nucleotide polymorphism
UTR: Untranslated region
