# Erratum to: Exome genotyping, linkage disequilibrium and population structure in loblolly pine (*Pinus taeda* L.)

**DOI:** 10.1186/s12864-016-3220-2

**Published:** 2016-11-04

**Authors:** Mengmeng Lu, Konstantin V. Krutovsky, C. Dana Nelson, Tomasz E. Koralewski, Thomas D. Byram, Carol A. Loopstra

**Affiliations:** 1 Department of Ecosystem Science and Management, Texas A&M University, 2138 TAMU, College Station, TX 77843-2138 USA; 2 Molecular and Environmental Plant Sciences Program, Texas A&M University, 2474 TAMU, College Station, TX 77843-2474 USA; 3 Department of Forest Genetics and Forest Tree Breeding, Georg-August-University of Göttingen, Göttingen, 37077 Germany; 4 N. I. Vavilov Institute of General Genetics, Russian Academy of Sciences, Gubkina Str, Moscow, 119333 Russia; 5 Genome Research and Education Center, Siberian Federal University, 50a/2 Akademgorodok, Krasnoyarsk, 660036 Russia; 6 USDA Forest Service, Southern Research Station, Southern Institute of Forest Genetics, 23332 Success Road, Saucier, MS 39574 USA; 7 University of Kentucky, Forest Health Research and Education Center, 730 Rose Street, Lexington, KY 40546 USA; 8 Texas A&M Forest Service, 2585 TAMU, College Station, TX 77843-2585 USA

## Erratum

The accession number for the sequence submitted to NCBI Sequence Read Archive (SRA) listed in the original article [1] is incorrect; SRP075763 should be SRP075363.
