# Supplementary figures and images for: Exome genotyping, linkage disequilibrium and population structure in loblolly pine (Pinus taeda L.)
Source: BMC Genomics. 2016 Sep 13;17(1):730. doi: 10.1186/s12864-016-3081-8 (PMC5022155; doi:10.1186/s12864-016-3081-8)

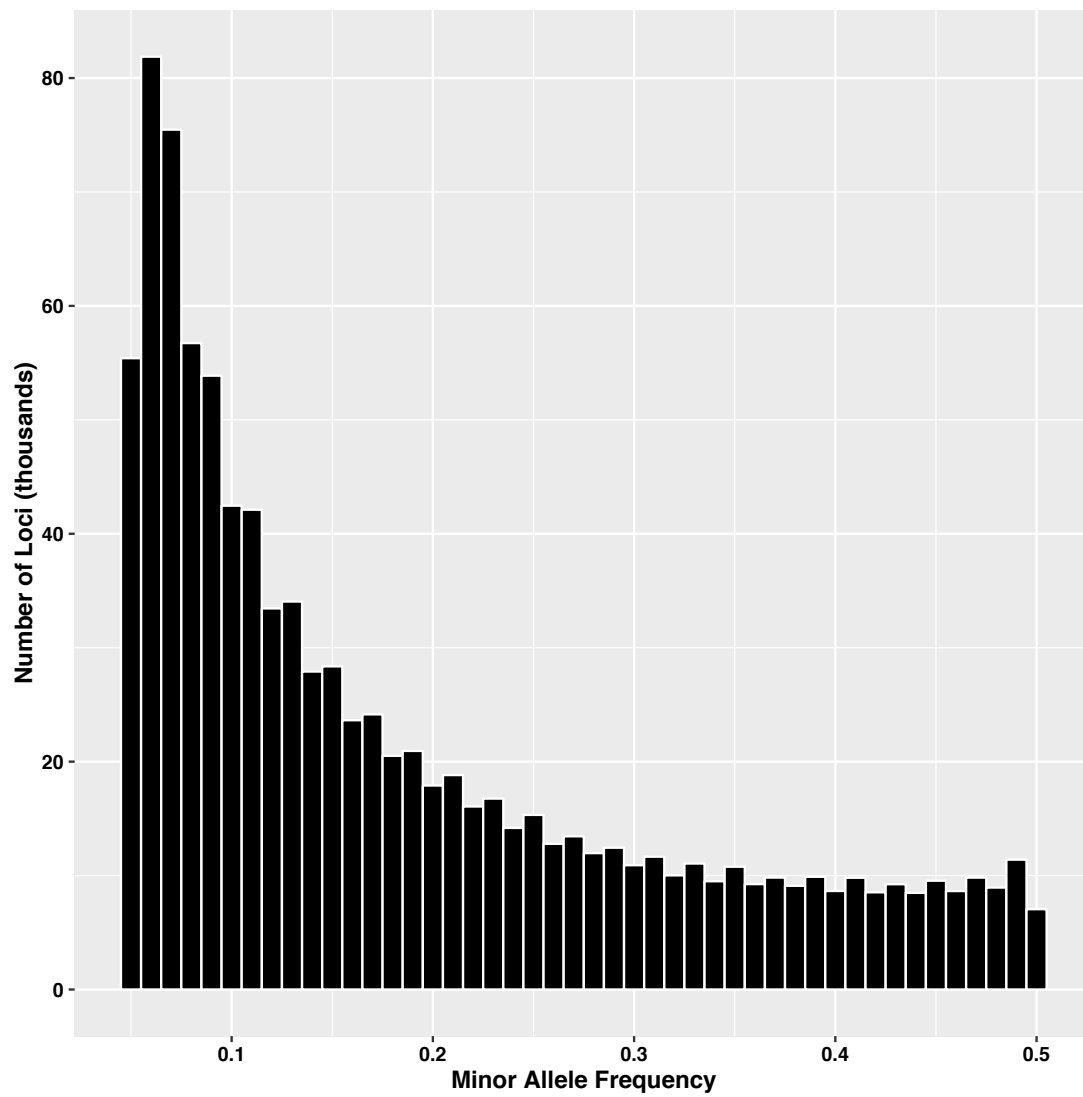

**Fig. S1** Minor allele frequency (MAF) distribution among 972,720 SNPs

Supplement: Additional file 2: Figure S1. — Minor allele frequency (MAF) distribution among 972,720 SNPs. (PDF 53 kb) [file 12864_2016_3081_MOESM2_ESM.pdf]
