# Supplementary material for: Exome genotyping, linkage disequilibrium and population structure in loblolly pine (Pinus taeda L.)
Source: BMC Genomics. 2016 Sep 13;17(1):730. doi: 10.1186/s12864-016-3081-8 (PMC5022155; doi:10.1186/s12864-016-3081-8)
Supplement: Additional file 3: Table S2. — Transition (TS) and transversion (TV) nucleotide substitutions summary. Numbers of TS and TV for 972,720 SNPs in different genomic regions. (PDF 51 kb) [file 12864_2016_3081_MOESM3_ESM.pdf]

Table S2 Transition (T_S) and transversion (T_V) nucleotide substitutions summary. Numbers of T_S and T_V for 972,720 SNPs in different genomic regions

**Substitution type**	**Total**	**CDS**	**Exon**	**5' UTR**	**3' UTR**
AC	89870	46655	52192	1834	3538
AG	320946	170211	186063	5388	9913
AT	63816	31523	36121	1341	3139
CG	85174	47396	52783	1871	3360
CT	322874	171247	187210	5438	9983
GT	90040	46620	52281	1821	3654
T_s	643820	341458	373273	10826	19896
T_v	328900	172194	193377	6867	13691
