# Supplementary material for: Exome genotyping, linkage disequilibrium and population structure in loblolly pine (Pinus taeda L.)
Source: BMC Genomics. 2016 Sep 13;17(1):730. doi: 10.1186/s12864-016-3081-8 (PMC5022155; doi:10.1186/s12864-016-3081-8)
Supplement: Additional file 4: Table S3. — Nucleotide diversity (π) estimated in a sliding window of 50 bp with a step of 25 bp in different genomic regions. (PDF 59 kb) [file 12864_2016_3081_MOESM4_ESM.pdf]

Table S3 Nucleotide diversity (π) estimated in a sliding window of 50 bp with a step of 25 bp in different genomic regions

**Genomic region**	**Range**	**Mean**	**Median**
Total	0.0016 – 0.1204	0.0119	0.0092
CDS	0.0016 – 0.1204	0.0117	0.0090
Exon	0.0016 – 0.1204	0.0116	0.0090
5’ UTR	0.0018 – 0.0787	0.0104	0.0083
3’ UTR	0.0017 – 0.0827	0.0100	0.0081
On Annotated Genes	0.0016 – 0.1204	0.0117	0.0090
Out of Annotated Genes	0.0016 – 0.1087	0.0122	0.0095
