# Supplementary material for: Exome genotyping, linkage disequilibrium and population structure in loblolly pine (Pinus taeda L.)
Source: BMC Genomics. 2016 Sep 13;17(1):730. doi: 10.1186/s12864-016-3081-8 (PMC5022155; doi:10.1186/s12864-016-3081-8)
Supplement: Additional file 5: Figure S2. — F ST distribution across all loci. The range is between -0.01 and 0.72, with a median of 0.0087. The mean F ST is 0.026, and the weighted F ST is 0.028. (PDF 59 kb) [file 12864_2016_3081_MOESM5_ESM.pdf]

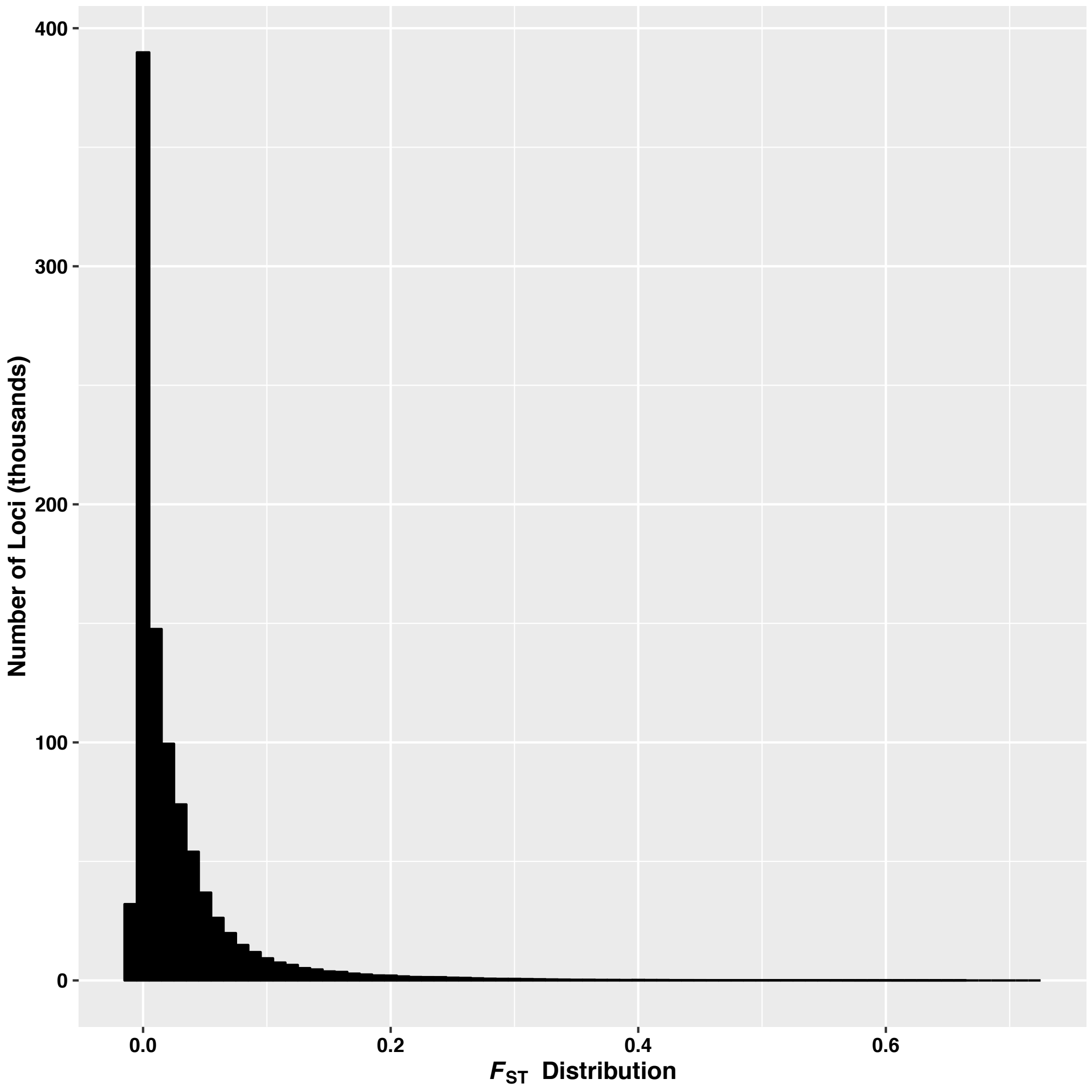

Fig. S2 F_{ST} distribution across all loci. The range is between -0.01 and 0.72, with a median of 0.0087. The mean F_{ST} is 0.026, and the weighted F_{ST} is 0.028
